# An Integrative Literature Review of Organisational Factors Associated with Admission and Discharge Delays in Critical Care

**DOI:** 10.1155/2015/868653

**Published:** 2015-10-19

**Authors:** Laura-Maria Peltonen, Louise McCallum, Eriikka Siirala, Marjaana Haataja, Heljä Lundgrén-Laine, Sanna Salanterä, Frances Lin

**Affiliations:** ^1^Department of Nursing Science, University of Turku, 20520 Turku, Finland; ^2^ICU, TG3B, Turku University Hospital, Hospital District of Southwest Finland, Kiinamyllynkatu 4-8, 20520 Turku, Finland; ^3^School Nursing & Midwifery, University of Dundee, 11 Airlie Place, Dundee DD1 4HJ, UK; ^4^School of Health, Nursing & Midwifery, University of West of Scotland, Ayr, Ayrshire KA8 0SX, UK; ^5^Intensive Care Unit, Operative Profit Centre, Oulu University Hospital, P.O. Box 21, 90029 Oulu, Finland; ^6^Hospital District of Southwest Finland, Administrative Centre, Bureau of Nursing Care, P.O. Box 52, 20521 Turku, Finland; ^7^School of Nursing and Midwifery, Centre for Health Practice Innovation, Menzies Health Institute Queensland, Griffith University, Gold Coast Campus, Gold Coast, QLD 4222, Australia

## Abstract

The literature shows that delayed admission to the intensive care unit (ICU) and discharge delays from the ICU are associated with increased adverse events and higher costs. Identifying factors related to delays will provide information to practice improvements, which contribute to better patient outcomes. The aim of this integrative review was to explore the incidence of patients' admission and discharge delays in critical care and to identify organisational factors associated with these delays. Seven studies were included. The major findings are as follows: (1) explanatory research about discharge delays is scarce and one study on admission delays was found, (2) delays are a common problem mostly due to organisational factors, occurring in 38% of admissions and 22–67% of discharges, and (3) redesigning care processes by improving information management and coordination between units and interdisciplinary teams could reduce discharge delays. In conclusion, patient outcomes can be improved through efficient and safe care processes. More exploratory research is needed to identify factors that contribute to admission and discharge delays to provide evidence for clinical practice improvements. Shortening delays requires an interdisciplinary and multifaceted approach to the whole patient flow process. Conclusions should be made with caution due to the limited number of articles included in this review.

## 1. Introduction

Efficient care processes demand appropriate coordination of human and material resources to meet patients' needs in critical care. Nonetheless, coordination of these resources presents a challenge given the interdisciplinary nature of intensive care therapy and the fact that critically ill patients' clinical conditions can change rapidly [1–6].

Current research shows that intensive care unit (ICU) admission delays are associated with increased ICU mortality [7, 8], increased in-hospital mortality [8–12], increased hospital length of stay [8], increased requirement for respiratory support [13], and longer ventilator care time [13]. Furthermore, every hour of ICU admission delay may increase the risk of death by 1.5% [14].

The optimal timing of an ICU discharge is important because early discharge and discharge delays are associated with an increased mortality [15]. Higher ICU bed occupancy is associated with an increased risk of death [16] and an increased risk of ICU readmission [16, 17]. This is supposedly due to overload of the ICU capacity, which may impact physician decision-making and result in premature discharge from the ICU [16]. Also, night-time discharges from the ICU are associated with increased mortality and the reason for this might be that the patients are not fully ready to be discharged and that they have a higher Acute Physiology and Chronic Health Evaluation (APACHE II) score [2]. One study found that 18% (*n* = 153) of the ICU discharges were unsuccessful and exceeded 24 hours and that 21% (*n* = 32) of these were due to a request by a clinician because of disagreement between ICU and ward staff, regarding the medical suitability of the discharge, related to the ability to care for the patient on the ward [18]. This study reported further that 46% (*n* = 70) of the unsuccessful discharges were due to administrative difficulties (e.g., no available bed on the ward and disagreement where the patient should be transferred for further care).

There have been attempts to improve admission and discharge delays of critically ill patients. Interventions include changes to improve patient flow from the emergency department (ED) to the ICU [19] and direct admissions from the emergency medical services (EMS) to a coronary care unit to reduce admission delays of myocardial infarction patients [20, 21]. Other interventions such as ICU liaison and outreach services have had an advantageous consequence on ICU discharge delays [22]. However, a comprehensive review on the incidence, the causes, the costs, and interventions aimed at reducing admission and discharge delays in critical care is still lacking. With critical care we refer to health care that is provided to a critically ill patient during a medical emergency or a crisis. In this review we focus on critical care given in an ICU or a high dependency unit (HDU).

The conceptual framework used here is based on work by Vincent et al. (1998) [34]. They identified six factors that influence clinical practice: institutional context (e.g., economic and regulatory context), organisational and management factors (e.g., financial resources and constraints), work environment (e.g., staffing levels and skills mix), team factors (e.g., communication and team structure), individual factors (e.g., knowledge and skills), task factors (e.g., task design), and patient characteristics (e.g., condition).

Admission and discharge delays are common in the care process for critically ill patients and delays may be caused by different factors. Factors about patient characteristics, such as a deteriorating physical condition, are difficult to eliminate through process improvements. However, organisational factors which may delay care processes could be improved. Based on the conceptual framework presented above, we included organisational and management factors, work environment factors, team factors, individual factors, and task factors under the broad umbrella term of organisational factors in this review. Identifying the reasons for delays caused by organisational factors would enable improvements in care processes and avoid bed blocks. This could ultimately improve patient flow through the hospital, enhance quality of patient care, and ensure better care outcomes.

The aim of this review is to explore the incidence of patients' admission and discharge delays in critical care, including the ICU and the HDU, and to identify the organisational factors that are associated with these delays. Here, we use the term ICU for both the ICU and the HDU. The questions directing this integrative review are

1. What is the incidence of ICU admission and discharge delays?
2. What are the contributing organisational factors for delayed admissions and discharge delays to and from the ICU after the physician has made the decision to admit or discharge a patient?
3. How can ICU admission and discharge delays be minimised?

## 2. Methods

### 2.1. Design

An integrative literature review was conducted. The integrative review method allows the inclusion of both qualitative and quantitative studies [35, 36], which can extend the generalisability of the results [36–38]. The rigour of this review was addressed through the development of a review protocol [35, 39]. This was based on the five phases identified by Whittemore and Knafl [39]. These include (1) problem identification and defining the key concepts, (2) database search with inclusion or exclusion of studies, (3) evaluation of studies, (4) data extraction from individual studies, and (5) data analysis and synthesis.

In this review, “population” was defined as critical care patients, including intensive care and high dependency care patients. The “phenomenon of interest” was defined as organisational factors, which contribute to patient admission or patient discharge. The “outcomes of interest” were defined as admission delays and discharge delays to and from the ICU or HDU.

We searched five databases including PubMed, Cinahl, Scopus, the Web of Science, and the Cochrane library. Database-specific terms (e.g., MeSH-terms) were used in the search process where possible. Free text terms were limited to title, abstract, or keywords. The search was also limited to peer-reviewed journals. The search was done in two steps by two investigators. The first broad search was carried out to find appropriate concepts and key terms; for the second search, these key terms were used to search for papers to be included. A librarian was consulted. No language or date restriction was used. The reference lists of the included articles were screened but no additional paper was found.

The used search terms were “intensive care,” “intensive care unit,” “critical care,” “ICU,” “high dependency unit,” “HDU,” “patient admission,” “patient discharge,” “patient readmission,” “patient transfer,” “admission,” “discharge,” “transfer,” “readmission,” “handoff,” “handover,” “decision making,” “decision making, organizational,” “decision making, computer assisted,” “information management,” “management information systems,” “information access,” “medical informatics,” “nursing informatics,” “informatics,” “knowledge management,” “information flow,” “communication,” “hospital communication systems,” “communication barriers,” “delay,” “process assessment,” “health services research,” “outcome and process assessment,” “quality of care research,” “summative evaluation research,” “evaluation research,” “evaluation,” “organization and administration,” “meaningful use,” “centralized hospital services,” “assessment,” “waiting time,” “patient flow,” “continuity of patient care,” and “care process”.

### 2.2. Article Inclusion and Exclusion Criteria

A protocol for the selection of articles was developed based on the research questions. Each step of the selection process was completed by a minimum of two investigators. We included articles that focused on organisational factors associated with admission and discharge delays in critical care that were restricted to ICUs and HDUs, which provide care to critically ill adult patients. We excluded papers that focused on the ED, the post-anaesthesia care unit (PACU) and direct transfer from the EMS to the ICU. All articles that included ethical considerations for prioritising care were also excluded as were articles that focused on patient characteristics related issues as the main reason for the delay.

The specific inclusion criteria for the articles were as follows: (1) the included studies were original research with no restriction on design; (2) the type of participants included adult patients in intensive care, critical care, and high dependency; (3) the focus of the studies was on organisational factors (i.e., health service research) connected with patients' admission and discharge delays in critical care; and (4) the types of outcomes were patient admission delays, patient transfer delays, and patient discharge delays. The exclusion criteria hierarchy was as follows: (1) the outcomes were not about admission or discharge process; (2) the allocation was not about critical care; (3) the focus was not on decision-making concerning care coordination; and (4) the participants were children or neonates.

### 2.3. Search Results

**Figure 1:**
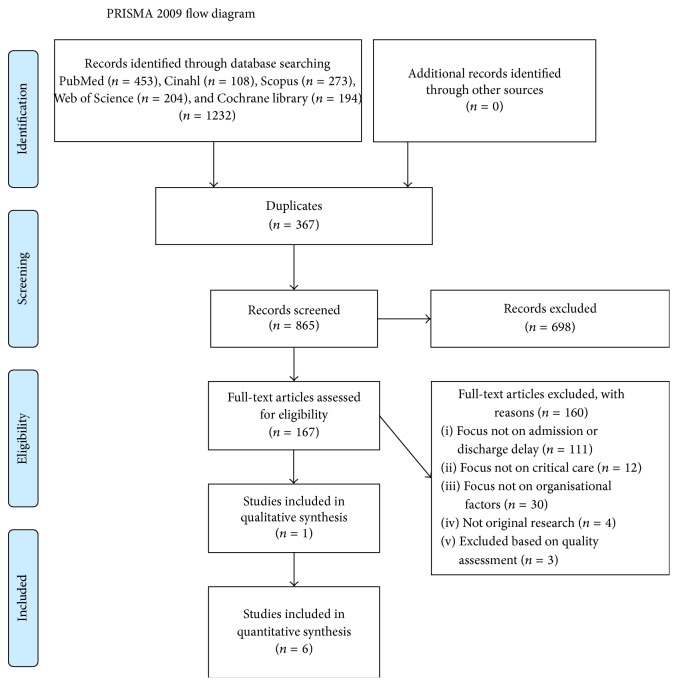
PRISMA flow diagram of the article selection process. From [23]. For more information, visit http://www.prisma-statement.org/.

The article selection process resulted in ten studies. Six studies were conducted in Australia, three in the USA, and one in the United Kingdom. Two of these explored ICU admission and eight ICU discharge processes. Four of the studies reported descriptive observational designs and six explanatory designs. However, three of these were excluded following the quality appraisal process, which is detailed in the next section. The selection process is described in detail in the PRISMA flow diagram in Figure 1.

### 2.4. Article Quality Assessment

Three evaluators independently assessed the quality of the studies using a two-piece assessment instrument by Kmet et al. 2004 [40]. Kmet et al. [40] recommended using a respective assessment tool for the assessing of qualitative studies and quantitative studies. These tools included lists of criteria, which had defined principles with four scoring options: yes, partially, no, and not applicable. The criteria are presented in Table 1. A summary score was calculated for each study by summing the total score and dividing it by the total possible score. Criteria that were not applicable were excluded from the sums.

**Figure 2:**
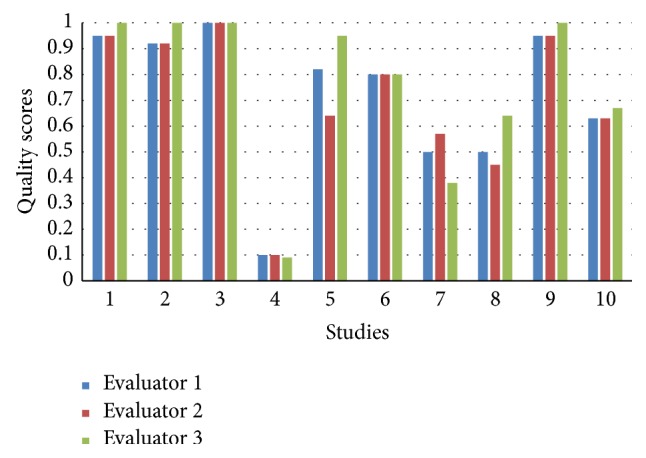
Results of quality assessment. Quality scores [0, 1] (*y*-axis): 0 = poor quality, 1 = excellent quality. Studies (*x*-axis): 1: Lin et al., 2013 [24]; 2: Chaboyer et al., 2012 [25]; 3: Chaboyer et al., 2006 [26]; 4: Crocker and Keller, 2005 [27]; 5: Gillman et al., 2006 [28]; 6: Johnson et al., 2013 [29]; 7: Kibler and Lee, 2011 [30]; 8: Silich et al., 2012 [31]; 9: Williams and Leslie, 2004 [32]; 10: Williams et al., 2010 [33].

The study selection process resulted in ten studies, which were advanced to the quality appraisal phase. Nine studies [25–33] were evaluated using the quantitative checklist and one study [24] was evaluated using the qualitative checklist. The agreement between the evaluators was calculated using the intraclass correlation coefficient (ICC) [41] with a two-way mixed model using the IBM SPSS Statistics version 22. A moderation process was used to ensure a clear understanding on how to use the tools. A consensus concerning the guidelines was reached. The ICC was calculated and this result was excellent (ICC = 0.981, 95% CI 0.946–0.995, *p* < 0.001). The results of the quality assessment are described in Figure 2.

The quality of the ten studies varied from 0.1 to 1, with seven studies rated by all of the evaluators as being above 0.55 (Figure 2). According to the guidelines for using the tool, studies with a quality score ranging from 0.55 (liberal) to 0.75 (conservative) and above can be included [40]. Seven articles above 0.55 were included in the review.

### 2.5. Data Extraction and Analysis

Two templates for data extraction were developed for the qualitative and quantitative studies. Three investigators extracted data independently from the studies to reduce the risk of transcription error. A meta-analysis was not appropriate due to the heterogeneity of research designs, methodologies, interventions, and instruments of the studies included within this review. Integrative reviews require a narrative analysis [37] and, therefore, narrative analysis was used to enable both the quantitative and qualitative data extracted from the studies to be summarised. Data about factors associated with ICU admission and discharge delays were synthesised using content analysis. Reporting of this study was guided by the requirements for reporting integrative reviews recommended by Evans [35].

## 3. Results

Seven studies were included within this review. One study was a qualitative study and six were quantitative studies. Six of the studies focused on discharge delays and one on admission delays. Four of the studies were observational, aiming at describing the causes and the factors connected with admission and discharge delays, while three of the studies were interventional, aiming at assessing the effect of an intervention on discharge delays. Based on the questions guiding this integrative review, the findings were organised into three topic areas. The topics include (1) the incidence and costs of ICU admission and discharge delays, (2) the causes of ICU admission and discharge delays, and (3) the interventions conducted to improve ICU admission and discharge delays. The studies included in the review are summarised in Table 2.

### 3.1. Incidence and Costs of ICU Admission and Discharge Delays

The frequency and time of the discharge delays varied between studies. Only one study exploring the admission delays was identified and included in this review. In this study the admission delays measured varied from less than 20 minutes to more than 2 hours [28], while the discharge delays measured ranged from 10 minutes to 26 days in the six other studies that were included in this review [24–26, 29, 32, 33]. The findings showed that 38% of ICU admissions were delayed [28]. The smallest incidence of delays in discharges (22%) occurred in a study where delay was measured in full days [29] and the largest (67%) occurred in a study where delay was measured starting from 2 hours [26]. The delays were mostly due to organisational factors, admission delays accounting for 65% [28] and discharge delays from 56% in one study [33] to 99% in another [29]. No cost calculations for the admission delays were provided, but one study estimated that the extra costs associated with the discharge delays amounted to US $21,547 per week in a 900-bed tertiary hospital with a 20-bed surgical ICU [29].

### 3.2. Contributing Factors of ICU Admission and Discharge Delays

The contributing factors for admission and discharge delays were related to information management and teamwork [24–26] and a lack of resources [24, 28, 29, 32, 33]. Bed availability issues were the most common reason for transfer delays [24, 28, 29, 32, 33]. The organisational contributing factors to ICU admission and discharge delays are presented in Table 3.

The discharge processes involved many disciplines, such as clerks, nurses, orderlies, and physicians on different position levels from different units in the hospitals, such as the bed management office, the ICU, and the wards. Contributing factors to discharge delays were conflicting goals among team members, teamwork issues [24], and communication breakdowns in the admission and discharge processes [24, 25]. Communication issues complicated the discharge processes and were associated with delays as staff from different units (between ICU and medical and surgical units) had different understanding about the discharge process and prioritised their work differently [24]. It was found that a shared situational awareness was associated with timely discharges and effective discharge processes [24–26].

A lack of resources including staff shortages and bed availability issues [24, 28, 29, 32, 33] and a high hospital census [29] were also contributing factors to admission and discharge delays. The discharge delays caused a bottleneck effect that prevented admission to the ICU [29]. This resulted in bed blocks, which was partly due to competing demands of medical staff. Such demands included excessive paperwork and other procedures on the ward [24]. Also, specific bed placement requirements were associated with the discharge delays. For example, patients with a multidrug resistant infection needed to be isolated in single patient rooms or rooms with patients with the same microbe infection [24, 29]. A lack of single patient rooms within the hospital hindered the placement of these patients and contributed to the discharge delays causing a bottleneck, which in turn impeded admission to the ICU [29]. Correspondingly, a high hospital census, with >95% occupancy, correlated with increased discharge delays [29]. ICU discharge delays were predicted by high patient acuity and discharge destination [32, 33]. One study concluded that improvements in bed management are essential for decreasing discharge delays [32]. Delayed patients were also more likely to be transferred during the night [29, 32] and weekends [32, 33]. This was attributed to the pressure that the ED places on the wards [32].

### 3.3. Interventions Conducted to Improve ICU Admission and Discharge Delays

We did not find any studies reporting interventions that decrease or minimise ICU admission delays. However, three studies reported on interventions aiming to decrease discharge delays [25, 26, 33]. Interventions to improve discharge delays were aimed at information management and coordination of the discharge process. Three studies also concluded that a coordinated hospital wide approach to bed management processes is important to decrease discharge delays in critical care areas [24, 25, 33].

Nursing and medical handovers did not support the discharge process sufficiently [24, 26] and cognitive and communication tools were implemented to improve information transfer between professionals and units to support clinical and administrative information management [24–26]. Likewise, several attempts existed to improve discharge processes through better communication between units [25] and liaison with ward staff before and after discharge [26]. Interdisciplinary communication meetings about available beds and discharge planning improved coordination of patients and staffing in the ICU and the ward, and a supportive culture aided junior staff in a fluent discharge process [24]. Better coordination on the wards was associated with improved resource use and decreased discharge delays [24–26].

One study by Chaboyer et al. [26] explored the impact of an ICU liaison nurse's role in reducing discharge delays. This nurse was involved in assessing patients for transfer, coordinating transfers, and liaising with ward staff during discharge. The findings showed that patients whose discharge did not involve the liaison nurse were 2.5 times more likely to experience a delay of four hours or longer when compared to patients whose discharge involved the liaison nurse.

A further study by Chaboyer et al. [25] evaluated the impact of redesigning the ICU discharge process on delays. The changes arising through the redesigned process focused on information management and improved communication between the ICU and the receiving units. These changes included a change agent, a handover communication sheet, a notice from the ward staff when they could receive the patient, and an Alert Sheet that informed receiving units about possible upcoming discharges. The cumulative effect of these changes resulted in enhanced knowledge transfer, which contributed to an improved ability to plan for new patients and a smoother transfer process. The intervention improved the discharge process by reducing the average delay time by 3.2 hours.

A third study by Williams et al. [33] explored the hypothesis that an ICU outreach team could reduce ICU discharge delays. The study assessed a critical care outreach team and compared discharge delay data from 2000/2001 and 2008. The study found that the delays from the ICU increased to 31% in 2008 compared to 27% for 2000/2001. The results did not support the hypothesis. However, quality appraisal of this study indicates that there were flaws in the choice of study design, which may have contributed to the findings that Williams et al. [33] presented.

## 4. Discussion

The aim of this review was to explore the incidence of patients' admission and discharge delays in critical care, including the ICU and the HDU, and to identify the organisational factors that are associated with these delays. The major findings are as follows. First, explanatory research with specific interventions to reduce discharge delays is scarce and only one study on admission delays was found. Therefore, the findings concerning admission delays are limited and descriptive in nature. Second, patient admission and discharge delays are a common problem, occurring in 38% of admissions and 22–67% of discharges. These delays are mostly due to organisational factors, admission delays accounting for 65% [28] and discharge delays from 56% in one study [33] to 99% in another [29]. Third, redesigning care processes by improving information management and coordination between units and interdisciplinary teams can reduce discharge delays.

The conceptual framework by Vincent et al. (1998) [34] supported us to identify the factors, which contribute to ICU admission and discharge delays. Also, all the organisational factors that were identified to contribute to ICU admission and discharge delays in this review (presented in Table 3) are issues acknowledged as factors that influence clinical practice in this conceptual framework.

Patient outcomes can be improved through efficient and safe care processes. Reducing ICU admission and discharge delays is imperative in order to provide timely services to critically ill patients and improve patient outcomes. One study found that patients experiencing a delayed discharge had a higher hospital mortality rate compared to other patients [33], which has been supported by other researches [15]. Patients who were delayed were also more likely to be transferred in the evening and at night [29, 32], and night-time discharges have been associated with an increased mortality [2]. Patient admission and discharge delays are also a costly problem. In particular, discharge delays are costly because the ICU cost per day is on average in the US $1,800 to $2,300 [42] and in European countries €1,168 to €2,025 [43].

The contributing organisational factors for admission and discharge delays were related to information management and teamwork [24–26] and a lack of resources [24, 28, 29, 32, 33]. The most common reason for transfer delays was bed availability issues [24, 28, 29, 32, 33]. Therefore, investments in hospital wide process improvements, including sufficient staffing, bed availability, and shared situational awareness through improved information management between units, have the potential to bring substantial savings through timely admissions and discharges and also directly improve patient outcomes. As a solution, we suggest not only an interdisciplinary approach but a transdisciplinary and multifaceted approach to the whole patient flow process because the decision-making processes in the admission and discharge processes are shared between professionals from different disciplines and units. The transdisciplinary approach reflects a deeper level of collaboration between team members in comparison to the interdisciplinary approach, and in the former professionals not only work together towards a joint goal but also generate the goals together [44, 45].

Research on patient admission delays in critical care is scarce; we did not find any studies explaining the relationship between organisational factors associated with admission delays. Discharge delays have been studied more often; we found three published interventional studies that aimed at reducing discharge delays. Based on our quality evaluation, one of these studies [33] had a design that did not explain the relationships in the hypothesis as data was collected in two time points first in 2000/2001 and second in 2008 after the introduction of an outreach role. This timeframe makes it difficult to control for confounding. Therefore, we only used descriptive data from this study. The two other studies found that redesigning the discharge process and introducing a new liaison nurse role by taking into account different elements of the discharge process were effective in reducing discharge delays [25, 26]. In light of two studies with convergent results [25, 26], it seems that interdisciplinary interventions focusing on improving the ICU discharge process may decrease discharge delays in critical care.

We located only seven studies to include in the review. These included only one qualitative study, and therefore no metasynthesis of qualitative study findings was possible. Furthermore, due to the small number of experimental studies, no meta-analysis was possible either. Three other studies that aimed at reducing admission and discharge delays to or from the ICU were excluded based on the quality assessment.

Only one study included in this review used a conceptual framework [24] and there is a clear lack of consensus around how admission and discharge delays are defined and measured in different studies. This might explain the variation in the reported incidences of discharge delays. All the studies were single-centre studies and most of them were conducted in Australia. The studies clearly concluded that more research is needed to improve delays. Therefore, we suggest an international research approach to explore the situation in other countries as well as to develop and test theories, which aim at a shared understanding. This will help complex interventions to be designed that effectively address the identified problems to improve admission and discharge delays in critical care.

There are several limitations to this review. First, we excluded all articles focusing mainly on the ED when selecting the articles based on our research question. Therefore, a selection bias may exist concerning studies on ICU admission delays, as these delays could have been reported as ED discharge delays. Further, we identified only one study exploring ICU admission delays and therefore the findings in this review concerning ICU admission delays are purely descriptive. Second, the quality of the articles included in this review is modest. Furthermore, few explanatory designs were found and all of the studies included in the review were single-centre studies with no generalisable results. We included all studies with a quality score above 0.55, which was defined in the quality evaluation guidelines by Kmet et al. [40]. Based on our experience, the lower limit of 0.55, as suggested as a liberal cut-point in the quality assessment guidelines for including studies in the review, is too low because studies with such a low quality score have a large risk of bias due to limitations in different aspects of the conducted research. These aspects are presented as quality evaluation criteria in Table 1. Therefore, we propose in the future to use the upper limit of 0.75 when including studies in reviews in order to reduce the risk of bias. This cut-point related finding is supported by a more recent study [46]. Furthermore, the evaluation instrument developed by Kmet et al. [40] was applicable for evaluating studies with different designs in critical care service research, but each evaluator may understand the criteria differently. Hence, when using this tool the evaluators need to discuss their understanding of the criteria and the scoring before starting to evaluate the studies. Third, the estimated costs were extracted from only one study conducted in the USA for discharge delays; therefore, this figure is not generalisable. Nonetheless, it gives some estimate of the high cost of discharge delays. Fourth, due to the large variability in the reported delay times, we were unable to calculate an average cost for ICU discharge delays. However, it is evident that reducing ICU admission and discharge delays will improve patient outcomes and reduce health care costs.

## 5. Conclusion

In conclusion, decreasing admission and discharge delays in critical care is essential because such delays have a serious impact on patient outcomes and healthcare costs. To date, limited research has considered ICU admission delays, and only a few studies have assessed ICU discharge delays. The findings concerning ICU admission delays are descriptive due to the fact that only one study exploring ICU admission delays was included in this review, and, therefore, conclusions about these findings must be made with caution. Most of the research on this topic is observational and descriptive and only a few explanatory studies exist concerning ICU discharge delays. However, they are single-centre studies, and therefore the results are not generalisable. Based on the findings presented in this integrative literature review, the following organisational factors are associated with ICU admission and discharge delay: information management and teamwork (including conflicting goals, teamwork issues, communication breakdowns, and lack of shared situational awareness) and a lack of resources (including a busy workload, lack of an available bed, specific bed placement requirements, lack of adequate staff, the receiving unit being not ready for transfer, and the time of discharge, i.e., night and weekend transfers).

Reducing ICU admission and discharge delays requires an interprofessional and multifactorial evaluation approach to the whole critical care process in the hospital due to the multiple actors and disciplines involved in the provision of critical care. This finding is supported by several studies, for example, [3, 25, 33]. Also, stronger and generalisable evidence is needed based on multicentre studies of interventions to reduce ICU admission and discharge delays.

Future research in critical care is also needed to evaluate admission and discharge processes; to explain the relationships between factors associated with admission and discharge delay; to ensure the effect of the existing interventions to reduce discharge delays; to find new interventions to reduce delays and to improve bed availability, which is also stated in several studies [24, 29, 32, 33]; to explore the use of admission and discharge guidelines and adherence to these; and to determine the exact costs of the ICU admission and discharge delays.

The findings of this review can be used to develop research questions to explain relationships between factors associated with ICU admission and discharge processes as a means of decreasing admission and discharge delays. The findings may also be used for process improvements in the critical care setting to improve care and patient outcomes. It is evident that, due to the complexity of the critical care process, more research is needed to explain relationships between the various elements in the admission and discharge processes.

## Figures and Tables

**Table 1:** Criteria for quality evaluation by Kmet et al. 2004 [40, pages 4-5].

Criteria used for quantitative studies	Criteria used for qualitative studies
(1) Question/objective sufficiently described? (2) Study design evident and appropriate? (3) Method of subject/comparison group selection or source of information/input variables described and appropriate? (4) Subject (and comparison group, if applicable) characteristics sufficiently described? (5) If interventional and random allocation was possible, was it described? (6) If intervention and blinding of investigators were possible, was it reported? (7) If interventional and blinding of subjects were possible, was it reported? (8) Outcome and (if applicable) exposure measure(s) well defined and robust to measurement/misclassification bias? Means of assessment reported? (9) Sample size appropriate? (10) Analytic methods described/justified and appropriate? (11) Some estimate of variance is reported for the main results? (12) Controlled for confounding? (13) Results reported in sufficient detail? (14) Conclusions supported by the results?	(1) Question/objective sufficiently described? (2) Study design evident and appropriate? (3) Context for the study clear? (4) Connection to a theoretical framework/wider body of knowledge? (5) Sampling strategy described, relevant and justified? (6) Data collection methods clearly described and systematic? (7) Data analysis clearly described and systematic? (8) Use of verification procedure(s) to establish credibility? (9) Conclusions supported by the results? (10) Reflexivity of the account?

**Table 2:** Studies included in the review.

Authors, publication year, and country	Aim (A), study design (D), and intervention (I)	Setting (S) and sample size (SS)	Findings of interest in this review	Generalisation	Suggested future research
Chaboyer et al. [26], 2006, Australia	A: to examine the impact of an ICU liaison nurse on discharge delay D: prospective block intervention study I: liaison nurse in discharge process	S: 580-bed tertiary hospital with a 13-bed ICU SS: *n* = 186 patients (*n* = 101 control group, *n* = 85 intervention group)	The liaison nurse assessed patients for transfer to the ward and coordinated patient transfers, including communication with ward staff prior to and after discharge. Patients whose discharge did not involve the liaison nurse were 2.5 times more likely to have a delay of 4 h or longer in comparison to the ones that did	Single-centre study	A hospital-wide perspective on service enhancements Evaluate the liaison nurse's role Explore factors influencing ICU discharge delay

Chaboyer et al. [25], 2012, Australia	A: to evaluate the impact of an ICU nursing discharge process redesign D: time series design I: redesigned discharge process	S: 12-bed general ICU incl. HDU beds in a 580-bed hospital SS: *n* = 1,787 discharges (*n* = 1,001 before, *n* = 786 after implementation)	A redesigned ICU discharge process demonstrated a 3.2 h reduction in the average patient discharge delay time (from 4.6 h to 1 h). Both ward and ICU staff were involved in the process and this may have contributed to mutual situational awareness leading to more timely and effective discharge processes. The changes decreased discharge delays without increasing mortality or readmission rates	Single-centre study	Demonstrate the effect of different changes in the ICU discharge process on outcomes

Gillman et al. [28], 2006, Australia	A: to determine the incidence and nature of adverse events occurring during transfer from the ED to the ICU D: prospective observational study	S: ED and ICU in a tertiary hospital SS: *n* = 290 patient transfers	38% (*n* = 54) of patient transfers from the ED to the ICU were delayed. Delay times were less than 20 min (68%), 21–60 min (24%), and 61–120 min (9%) and more than 121 min (5%). Delays were caused by a lack of ICU beds (30%), a lack of staff (15%), busy workload in the ICU (9%), delay in the availability of transfer orderlies (11%), computed tomography (CT) not ready (9%), OR not ready for transfer (7%), ICU busy (5%), no anaesthetist (6%), speciality rereview (7%), and unknown reasons (6%).	Single-centre study	Establish benchmark indicators for adverse events and transfer delays

Johnson et al. [29], 2013, USA	A: to analyse the incidence, causes, and costs of delayed transfer from a surgical intensive care unit (SICU) D: prospective observational study	S: 900-bed tertiary hospital with a 20-bed surgical ICU SS: *n* = 731 patient transfers	22% (*n* = 160) of ICU transfers to the wards were delayed. Delay times varied from 1 to 6 days (mean 1.5, median 2 days). Delays were caused by lack of an available ward bed (71%), lack of an appropriate room for infectious patients (18%), change of medical speciality (7%), and lack of an available bedside nurse for the patient (3%). A positive association existed between the number of patients in the hospital and the number of ICU beds occupied by delayed patient transfers (Spearman rho = 0.27, *p* < 0.0001). Delayed patients were more likely to be transferred between 7 p.m. and 6.59 a.m. (21% versus 12%, *p* < 0.005). The delay-related costs were estimated to be US $21,547/week	Single-centre study	Calculate cost data for ICU discharge delays Analyse census of wards for delayed patients, while emphasising bed availability Interventions to reduce beds occupied by discharge-ready patients Study further ICU transfer delays

Lin et al. [24], 2013, Australia	A: to explore the factors influencing the ICU patient discharge process D: ethnographic study	S: 580-bed tertiary hospital with 14-bed, level 3 medical or surgical ICU SS: *n* = 28 discharges	43% of the ICU discharges were delayed. 11% of the delays lasted for 1 day, while 32% of delays lasted for 2-3 days. Altogether, 33% of discharges were delayed due to the limited availability of ward beds. Three activity systems were identified in the discharge process: the ICU discharge activity, the ward accepting the ICU patient, and the management of hospital beds. Better coordination, communication, and shared goals could decrease discharge delays	Single-centre study	Strategies to improve the efficiency of acute care beds Develop tools to support the discharge process Explore the role of the discharge guidelines

Williams and Leslie [32], 2004, Australia	A: to examine the prevalence and reasons for delayed discharges D: cross-sectional observational study	S: 955-bed tertiary hospital with a 22-bed mixed ICU SS: *n* = 652 discharges	27.3% (*n* = 176) of ICU discharges were delayed. The median delay time was 21.3 h with a variation ranging from 10 min to 26 days. Delays were caused by no available beds (75%), ward bed delayed (5.7%), medical complication (8.5%), the environment (0.6%), a lack of medical coverage (0.6%), transport (0.6%), unknown reasons (5.7%), the closure of a ward (1.7%), and other reasons (e.g., lack of an available room for infection control, no available nurses, and inadequate nursing skills on the ward) (1.7%). Most delays occurred on weekends	Single-centre study	Apt admission and discharge criteria Determine the effect of hospital occupancy on discharge and the factors causing care transition between medical specialties Determine the optimal number of ICU and intermediate care beds

Williams et al. [33], 2010, Australia	A: to examine whether the introduction of a critical care outreach role would decrease the frequency of ICU discharge delays D: comparison of observational data from 2000/2001 and 2008	S: 955-bed tertiary hospital with a 22-bed general ICU. SS: in 2000/2001 *n* = 607, in 2008 *n* = 516 discharges	31% (*n* = 488) of the ICU discharges were delayed by more than 8 h in 2008, with an increase of 6% from 2000/2001 (*p* < 0.001). In 2008, the reasons for delay included bed delay (17%), no bed (36%), staff shortage (2%), no accommodation (1%), other delays (20%), and medical concerns (24%). In 2000/2001, the reasons for delay included no bed (74%), bed delay (6%), medical reasons (9%), no accommodation (1%), and other reasons (10%). The mean delay time, when excluding medical reasons, was 21 h in 2000/2001 and 25 h in 2008. After-hour discharges were more frequent in delayed discharges and these occurred more often in 2008 when compared to 2000/2001. Patients also spent more time in the hospital when the discharge was delayed	Single-centre study	Examine the effects of bed management models on patient flow Study the associations with ICU discharge delays

**Table 3:** Organisational factors contributing to ICU admission and discharge delays.

Contributing factor	Admission	Discharge
Information management and teamwork		
Conflicting goals		[24]
Teamwork issues		[24]
Communication breakdowns		[24–26]
Lack of shared situational awareness		[24–26]
Lack of resources		
Busy workload (unit/hospital)	[28]	[24, 29, 32]
Lack of an available bed	[28]	[24, 29, 32, 33]
Specific bed placement requirements due to, for example, infection precautions		[29, 32]
Lack of adequate staff	[28]	[29, 32, 33]
Receiving unit not ready for transfer	[28]	[24]
Time of discharge (night and weekend transfers)		[29, 32, 33]

## References

[1] Brand S. L. (2006). Nurses' roles in discharge decision making in an adult high dependency unit. *Intensive and Critical Care Nursing*.

[2] Lin F., Chaboyer W., Wallis M. (2009). A literature review of organisational, individual and teamwork factors contributing to the ICU discharge process. *Australian Critical Care*.

[3] Lin F., Chaboyer W., Wallis M. (2014). Understanding the distributed cognitive processes of intensive care patient discharge. *Journal of Clinical Nursing*.

[4] Lundgrén-Laine H., Kontio E., Perttilä J., Korvenranta H., Forsström J., Salanterä S. (2011). Managing daily intensive care activities: an observational study concerning ad hoc decision making of charge nurses and intensivists. *Critical Care*.

[5] Miller A., Weinger M. B., Buerhaus P., Dietrich M. S. (2010). Care coordination in intensive care units: communicating across information spaces. *Human Factors*.

[6] Miller A., Buerhaus P. I. (2013). The changing nature of ICU charge nurses' decision making: from supervision of care delivery to unit resource management. *Joint Commission Journal on Quality and Patient Safety*.

[7] Bing-Hua Y. U. (2014). Delayed admission to intensive care unit for critically surgical patients is associated with increased mortality. *The American Journal of Surgery*.

[8] Chalfin D. B., Trzeciak S., Likourezos A., Baumann B. M., Dellinger R. P. (2007). DELAYED study group: impact of delayed transfer of critically ill patients from the emergency department to the intensive care unit. *Critical Care Medicine*.

[9] Chiavone P. A., Rasslan S. (2005). Influence of time elapsed from end of emergency surgery until admission to intensive care unit, on Acute Physiology and Chronic Health Evaluation II (APACHE II) prediction and patient mortality rate. *Sao Paulo Medical Journal*.

[10] Mokart D., Lambert J., Schnell D. (2013). Delayed intensive care unit admission is associated with increased mortality in patients with cancer with acute respiratory failure. *Leukemia & Lymphoma*.

[11] Parkhe M., Myles P. S., Leach D. S., Maclean A. V. (2002). Outcome of emergency department patients with delayed admission to an intensive care unit. *Emergency Medicine*.

[12] Phua J., Ngerng W. J., Lim T. K. (2010). The impact of a delay in intensive care unit admission for community-acquired pneumonia. *The European Respiratory Journal*.

[13] O'Callaghan D. J. P., Jayia P., Vaughan-Huxley E. (2012). An observational study to determine the effect of delayed admission to the intensive care unit on patient outcome. *Critical Care*.

[14] Cardoso L. T. Q., Grion C. M. C., Matsuo T. (2011). Impact of delayed admission to intensive care units on mortality of critically ill patients: a cohort study. *Critical Care*.

[15] Garland A., Connors A. F. (2013). Optimal timing of transfer out of the intensive care unit. *The American Journal of Critical Care*.

[16] Chrusch C. A., Olafson K. P., McMillan P. M., Roberts D. E., Gray P. R. (2009). High occupancy increases the risk of early death or readmission after transfer from intensive care. *Critical Care Medicine*.

[17] Baker D. R., Pronovost P. J., Morlock L. L., Geocadin R. G., Holzmueller C. G. (2009). Patient flow variability and unplanned readmissions to an intensive care unit. *Critical Care Medicine*.

[18] Levin P. D., Worner T. M., Sviri S. (2003). Intensive care outflow limitation—frequency, etiology, and impact. *Journal of Critical Care*.

[19] Howell E., Bessman E., Marshall R., Wright S. (2010). Hospitalist bed management effecting throughput from the emergency department to the intensive care unit. *Journal of Critical Care*.

[20] Prasad N., Wright A., Hogg K. J., Dunn F. G. (1997). Direct admission to the coronary care unit by the ambulance service for patients with suspected myocardial infarction. *Heart*.

[21] Steg P. G., Cambou J.-P., Goldstein P. (2006). Bypassing the emergency room reduces delays and mortality in ST elevation myocardial infarction: the USIC 2000 registry. *Heart*.

[22] Endacott R., Eliott S., Chaboyer W. (2009). An integrative review and meta-synthesis of the scope and impact of intensive care liaison and outreach services. *Journal of Clinical Nursing*.

[23] Moher D., Liberati A., Tetzlaff J., Altman D. G. (2009). Preferred reporting items for systematic reviews and meta-analyses: the PRISMA statement. *PLoS Medicine*.

[24] Lin F., Chaboyer W., Wallis M., Miller A. (2013). Factors contributing to the process of intensive care patient discharge: an ethnographic study informed by activity theory. *International Journal of Nursing Studies*.

[25] Chaboyer W., Lin F., Foster M., Retallick L., Panuwatwanich K., Richards B. (2012). Redesigning the ICU nursing discharge process: a quality improvement study. *Worldviews on Evidence-Based Nursing*.

[26] Chaboyer W., Thalib L., Foster M., Elliott D., Endacott R., Richards B. (2006). The impact of an ICU liaison nurse on discharge delay in patients after prolonged ICU stay. *Anaesthesia and Intensive Care*.

[27] Crocker C., Keller R. (2005). Nurse-led discharge to the ward from high dependency: a service improvement project. *Intensive and Critical Care Nursing*.

[28] Gillman L., Leslie G., Williams T., Fawcett K., Bell R., McGibbon V. (2006). Adverse events experienced while transferring the critically ill patient from the emergency department to the intensive care unit. *Emergency Medicine Journal*.

[29] Johnson D. W., Schmidt U. H., Bittner E. A., Christensen B., Levi R., Pino R. M. (2013). Delay of transfer from the intensive care unit: a prospective observational study of incidence, causes, and financial impact. *Critical Care*.

[30] Kibler J., Lee M. (2011). Improving patient transfer between the Intensive Care Unit and the Medical/Surgical floor of a 200-bed hospital in southern California. *Journal for Healthcare Quality*.

[31] Silich S. J., Wetz R. V., Riebling N. (2012). Using Six Sigma methodology to reduce patient transfer times from floor to critical-care beds. *Journal for Healthcare Quality*.

[32] Williams T., Leslie G. (2004). Delayed discharges from an adult intensive care unit. *Australian Health Review*.

[33] Williams T. A., Leslie G. D., Brearley L., Leen T., O'Brien K. (2010). Discharge delay, room for improvement?. *Australian Critical Care*.

[34] Vincent C., Taylor-Adams S., Stanhope N. (1998). Framework for analysing risk and safety in clinical medicine. *British Medical Journal*.

[35] Evans D., Webb C., Roe B. (2008). Overview of methods. *Reviewing Research Evidence for Nursing Practice: Systematic Reviews*.

[36] Whittemore R. (2005). Combining evidence in nursing research: methods and implications. *Nursing Research*.

[37] Whittemore R., Webb C., Roe B. (2008). Rigour in integrative reviews. *Reviewing Research Evidence for Nursing Practice: Systematic Reviews*.

[38] Finfgeld-Connett D. (2010). Generalizability and transferability of meta-synthesis research findings. *Journal of Advanced Nursing*.

[39] Whittemore R., Knafl K. (2005). The integrative review: updated methodology. *Journal of Advanced Nursing*.

[40] Kmet L. M., Lee R. C., Cook L. S. (2004). *Standard Quality Assessment Criteria for Evaluating Primary Research Papers from a Variety of Fields*.

[41] Shrout P. E., Fleiss J. L. (1979). Intraclass correlations: uses in assessing rater reliability. *Psychological Bulletin*.

[42] Dasta J. F., McLaughlin T. P., Mody S. H., Piech C. T. (2005). Daily cost of an intensive care unit day: the contribution of mechanical ventilation. *Critical Care Medicine*.

[43] Tan S. S., Bakker J., Hoogendoorn M. E. (2012). Direct cost analysis of intensive care unit stay in four European countries: applying a standardized costing methodology. *Value in Health*.

[44] Vyt A. (2008). Interprofessional and transdisciplinary teamwork in health care. *Diabetes/Metabolism Research and Reviews*.

[45] Reilly C. (2001). Transdisciplinary approach: an atypical strategy for improving outcomes in rehabilitative and long-term acute care settings. *Rehabilitation Nursing*.

[46] Murtola L., Lundgrén-Laine H., Saranto K., Castrén M., Kuusela T., Hyrynsalmi S., Ojala S. (2014). Information management efforts in improving patient safety in critical care—a review of the literature. *Communications in Computer and Information Science*.

